# Genetic control of neuronal activity enhances axonal growth only on permissive substrates

**DOI:** 10.1186/s10020-022-00524-2

**Published:** 2022-08-17

**Authors:** Francina Mesquida-Veny, Sara Martínez-Torres, José Antonio Del Río, Arnau Hervera

**Affiliations:** 1 Molecular and Cellular Neurobiotechnology, Institute for Bioengineering of Catalonia (IBEC), Barcelona, Spain; 2 Department of Cell Biology, Physiology and Immunology, University of Barcelona, Barcelona, Spain; 3 Network Centre of Biomedical Research of Neurodegenerative Diseases (CIBERNED), Institute of Health Carlos III, Ministry of Economy and Competitiveness, Madrid, Spain; 4 Institute of Neuroscience, University of Barcelona, Barcelona, Spain

**Keywords:** Optogenetics, Chemogenetics, Regeneration, Permissive substrate, Neuronal activity

## Abstract

**Background:**

Neural tissue has limited regenerative ability. To cope with that, in recent years a diverse set of novel tools has been used to tailor neurostimulation therapies and promote functional regeneration after axonal injuries.

**Method:**

In this report, we explore cell-specific methods to modulate neuronal activity, including opto- and chemogenetics to assess the effect of specific neuronal stimulation in the promotion of axonal regeneration after injury.

**Results:**

Opto- and chemogenetic stimulations of neuronal activity elicited increased in vitro neurite outgrowth in both sensory and cortical neurons, as well as in vivo regeneration in the sciatic nerve, but not after spinal cord injury. Mechanistically, inhibitory substrates such as chondroitin sulfate proteoglycans block the activity induced increase in axonal growth.

**Conclusions:**

We found that genetic modulations of neuronal activity on both dorsal root ganglia and corticospinal motor neurons increase their axonal growth capacity but only on permissive environments.

**Supplementary Information:**

The online version contains supplementary material available at 10.1186/s10020-022-00524-2.

## Background

Following injury, damaged axons from the central nervous system (CNS) degenerate and are unable to regenerate, while surviving fibres have a limited capacity to sprout and to re-establish lost connections, leading to functional impairment (Fitch and Silver 2008).

This failure of CNS axons to regenerate after injury is partly attributed to a hostile CNS environment for growth (Richardson et al. 1980; Huebner and Strittmatter 2009; Cregg et al. 2014). The injury site is rich in growth inhibitory proteins including myelin-associated glycoproteins, and chondroitin sulfate proteoglycans (CSPG) while lacking trophic support for axon regeneration (Jones et al. 2003; Ohtake and Li 2015; Sami et al. 2020). The limited intrinsic regenerative capacity of adult CNS axons also contributes to the failure of axon regeneration after injury (Curcio and Bradke 2018; Mahar and Cavalli 2018; He and Jin 2016). Yet, in the last years several studies have approached different aspects of the CNS physiology to both boost the intrinsic regrowth capacity and overcome the extrinsic inhibition (Anderson et al. 2018; Wu et al. 2015).

During development, when CNS neurons still retain their intrinsic ability to regenerate after axotomy (Arlotta et al. 2005), neuronal activity critically determines their connectivity, particularly onto spinal targets (Friel et al. 2014). In this direction, neuronal activation has been explored to try to increase the intrinsic capacity for axon regeneration as well as to overcome the extrinsic inhibition. Practically, electrical stimulation has been shown to enhance regeneration of sensory axons after peripheral nerve or dorsal columns injury (Goganau et al. 2018; Udina et al. 2008) and sprouting of cortical axons into contralateral spinal cord grey matter after pyramidotomy (Carmel et al. 2010, 2013, 2014).

In order to gain insight on the actual mechanisms underlying these improvements, specifically and remotely activating neurons through opto- and chemogenetics, has become the state-of-the-art approach for tailored activity modulation (Deisseroth 2015; Sternson and Roth 2014).

In this direction, the current study explores the therapeutic potential of modulating neuronal activity of sensory (dorsal root ganglia (DRG)) and motor descending (corticospinal motor neurons (CSMN)) neurons, via opto- and chemogenetic stimulation in regulating axonal growth after injury.

We found that specific neuronal stimulation induced increased axonal growth capacity both in vitro and in vivo, and both in sensory and motor neurons, but only when in presence of permissive and trophic environments. Opto- or chemogenetic stimulation did not overcome the inhibitory signalling induced by molecules such as CSPGs.

## Methods

### Mice

B6.Cg-Tg(Thy1-COP4/EYFP)18Gfng/J (Thy1-ChR2) (Jackson Laboratories) (Arenkiel et al. 2007) or wild type (WT) C57BL/6J mice (Charles River Laboratories) ranging from 6–10 weeks of age were used for the experiments and were randomly divided into the different experimental groups. Mice were anaesthetized with isoflurane (5% induction, 2% maintenance) during surgeries. For DRG neuronal cultures adult Thy1-ChR2 mice were used. Embryonic day 16.5 (E16.5) OF1 pregnant females were purchased from Charles River Laboratories, and the embryos were used for neuronal cortical cultures. All procedures were approved by the Ethics Committee on Animal Experimentation (CEEA) of the University of Barcelona (CEEA approval #276/16 and 141/15).

### Dorsal root ganglia (DRG) neuronal culture

DRGs from adult Thy1-ChR2 mice were dissected, washed in cooled Hank’s balanced salt solution (HBSS; ThermoFisher Scientific), and enzymatically digested (5 mg/ml Dispase II (Merck) and 2.5 mg/ml Collagenase Type II (ThermoFisher Scientific) in DMEM (ThermoFisher Scientific)) for 45 min at 37 °C. Next, the DRGs were resuspended in DMEM:F12 (ThermoFisher Scientific) media supplemented with 10% heat-inactivated Fetal Bovine Serum (FBS; ThermoFisher Scientific) and 1× B27 (ThermoFisher Scientific) and were mechanically dissociated by pipetting. After centrifugation, the resulting single cells were resuspended in culture media (DMEM:F12 media with 1× B27 and penicillin/streptomycin (P/S; ThermoFisher Scientific)) and plated in glass-coverslips (3000–4000 cells/well) or in microfluidic devices (20.000 cells/device) pre-coated with 0.1 mg/ml poly-d-lysine (2 h, 37 °C; Merck) and 2 μg/ml laminin (over-night (O/N); ThermoFisher Scientific) at RT (room temperature)). An additional incubation with 5, 10 or 20 μg/ml CSPGs (Merck) was performed (2 h at 37 °C) in growth-inhibitory substrate experiments. Cells were allowed to grow for 24 h or for 8 days (in the case of microfluidic devices) at 37 °C in a 5% CO_2_ atmosphere.

### Neuronal cortical culture

E16.5 OF1 mice (Charles River) embryos were used for neuronal cortical cultures. Brains were extracted, washed in cooled 0.1M phosphate-buffered saline (PBS; ThermoFisher Scientific) containing 6.5 mg/ml glucose (Sigma Aldrich) (PBS-glucose) and the meninges excised. Both cortical lobes were dissected, sliced in a McIlwain II tissue chopper (Capdem Instruments) and trypsinized for 15 min at 37 °C. The suspension was inactivated with Normal Horse Serum (NHS; ThermoFisher Scientific), incubated with 0.025% DNAse (Roche) PBS-glucose for 10 min at 37 °C and mechanically dissociated. Single cells were spun down and resuspended in Neurobasal™ (ThermoFisher Scientific) medium supplemented with 2 mM glutamine (ThermoFisher Scientific), P/S, 6.5 mg/ml glucose, NaHCO_3_ (Merck), 1× B27 and NHS 5% and plated in poly-d-lysine (0.1 mg/ml) pre-coated microfluidic devices (see below) (150.000 cells/device) or in glass-bottom plates (200.000 cells/plate). One day after seeding, culture media was changed, and NHS was excluded from the new media. Cells were maintained at 37 °C in a 5% CO_2_ atmosphere and culture media was changed every 2 days.

### Microfluidic devices

The designs used for microfluidic devices were modifications of previously published devices (Sala-Jarque et al. 2020; Taylor et al. 2005). One of the devices consists in 4 reservoirs of 7 mm diameter, connected in pairs by a longitudinal compartment (cell body and axonal compartments) which are in turn interconnected by 100 microchannels (10 μm × 10 μm × 900 μm). The other used device presented a similar design and included a perpendicular channel to the microchannels, located at the center of these. The masters were produced using standard photolithography techniques at IBEC Microfab Space. Polydimethylsiloxane (PDMS; Dow) was used to prepare the devices by soft lithography, which were subsequently attached to glass bottom dishes using oxygen plasma treatment.

In the first device, cortical neurons were seeded in one of the larger compartments (cell body compartment) and allowed to extend their axons across the microchannels. In the case of the second device, which was used with DRG neurons, neurons were seeded in the central channel. Vacuum aspiration in the axonal compartment allowed complete axotomies (Sala-Jarque et al. 2020).

### In vitro lentiviral production and infection

LV-EF1α-hChR2(H134R)-EYFP-WPRE (LV-ChR2) produced in our laboratory were used to introduce ChR2 expression in primary cortical neurons. For LV production, 293-FT (ATCC) cells were simultaneously co-transfected with three plasmids: pMD2.G (VSV-G envelope expressing plasmid), psPAX2 (lentiviral packaging plasmid), and pLenti-EF1α-hChR2(H134R)-EYFP-WPRE (transfer plasmid) in Opti-MEM (ThermoFisher Scientific) using Lipofectamine 2000 Transfection Reagent (ThermoFisher Scientific). Six hours after transfection media was replaced for culture media (Advanced DMEM (ThermoFisher Scientific) supplemented with 10% FBS, 1% P/S and 0.5% glutamine). Media was recovered at 48 h and 72 h post-transfection, filtered and centrifuged at 1200*g* to remove debris. The supernatant was then centrifuged at 26.000 rpm for 2 h at 4 °C in a Beckman conical tube containing a sucrose cushion (20%) for purification, and the viral pellet was finally resuspended in PBS-1% BSA. Cortical neurons were infected at 4 days in vitro (DIV) for 24 h, and high levels of YFP fluorescence could be observed at 7DIV, indicating positive infection.

### In vitro optogenetic stimulation

Thy1-ChR2 DRG neurons or cortical neurons infected with LV-ChR2 were used for in vitro optogenetic stimulation experiments. A 470 nm emission LED array (LuxeonRebel™) under the control of a Driver LED (FemtoBuck, SparkFun) of 600 mA and a pulse generator PulsePal (OpenEphys) (Siegle et al. 2017) was used to deliver blue light to neuronal cultures. The optogenetic stimulation protocol consisted in 1 h of illumination at 20 Hz of frequency with 5–45 ms pulses, in 1 s ON-1 s OFF periods. In the case of DRG neurons, stimulation was applied 2 h after seeding. For cortical neurons, two different stimulation time-points after the axotomy were assessed: 30 min after axotomy and 6 h after axotomy. To assess neuronal activation, neurons were fixed at the end of the stimulation. Uninjured cortical neurons were stimulated in this experiment.

### In vitro chemogenetic stimulation

10 µM Clozapine *N*-oxide (CNO; Tocris) was added to DRG cultures 6 h after axotomy and left in the media until fixation (24 h after the axotomy).

### Corticospinal tract and dorsal columns axotomy

A fine incision to the skin between the shoulder blades of anaesthetized mice and subsequent muscle removal allowed thoracic vertebral column exposure. A T9 laminectomy was performed, and the dura mater was excised. In corticospinal tract injuries, approximately two thirds of the spinal cord were severed laterally with a scalpel. In dorsal column axotomies (DCA), a dorsal hemisection was conducted with fine forceps.

### Sciatic nerve crush (SNC)

The sciatic nerve was exposed after a small incision on the skin over the posterior hindlimb and blunt dissection of the gluteus maximus and the biceps femoralis. Fine forceps were used to carefully compress the nerve orthogonally 2 × 10 s. Animals were allowed to recover for 24 h, when were then sacrificed and the sciatic nerve and the sciatic DRGs (L4, L5, L6) were dissected and processed.

### In vivo optogenetic stimulation

An optic fiber cannula (1.25 mm, 0.22 NA; Thorlabs) was implanted in the motor cortex (M1) of Thy1-ChR2 transgenic mice by stereotaxic surgery (− 1 mm antero-posterior (AP), 1.5 mm lateral to Bregma, 0.5 mm deep, (hind limb innervation region)) prior to injury and was fixed to the cranium with screws and dental cement. Correct placement of the cannula was tested for each animal by assessing the induction of unidirectional rotatory locomotion caused by unilateral optical stimulation of the motor component of the hind limb; animals that did not show this response were excluded from the study. At 7 days post-injury (DPI) animals started receiving daily optogenetic stimulations consisting in 1 h of illumination with 470 nm blue light, at 10 Hz of frequency with 10 ms pulses, in 1sON-4sOFF periods, for 5 consecutive days, 2 resting days followed by 5 more days. The illumination was delivered through the optic fiber cannula which was coupled to a 470 nm wavelength LED source (M470F3, Thorlabs) controlled by a pulse generator (Pulse Pal) (Siegle et al. 2017). The control group went through the same procedures than the experimental group without receiving illumination.

### In vivo chemogenetic stimulation

The commercial AAV5-hSynhM3D(Gq)-mCherry or the control virus AAV5-hSyn-mCherry (Addgene) were injected into the sciatic nerve of C57BL/6J mice (1 µl/sciatic nerve) 4–5 weeks before the experiment. For chemogenetic stimulation, the animals received two daily intraperitoneal (i.p.) injections of 5 mg/kg CNO (Tocris). After DCA, injections were delivered starting from 3DPI and lasted 7 consecutive days. In the case of SNC, the injections were given prior to injury: from 3 days pre-injury to the same day of the injury (in total 4 days of chemogenetic stimulation).

### Behavioural assessment of sensorimotor function

Sensorimotor deficits and recovery were evaluated using the gridwalk and the BMS (Basso Mouse Scale) tests at − 1, 1, 7, 14, 21, 28 and 35 DPI for CST injuries and at − 1, 1, 3, 7, 10, 21 and 28 DPI for DCA. During the gridwalk animals were recorded while walking three times on a grid of 1 × 1 cm squares (total longitude of the grid: 50 cm). The number of missteps in relation to the total number of steps was blindly quantified. In BMS evaluations mice were allowed to freely move in an open field and a score was assigned to each of them according to the BMS scale (Basso et al. 2006). In brief, this scale grades from 0 to 9 the locomotor capacity of the hind limbs depending on several aspects such as ankle movement, paw placing and position or stepping.

### Tracing of injured spinal cords

A fluorescent tracer (10% Dextran-Alexa 594, 10.000 MW, ThermoFisher Scientific) was injected with a stereotaxic frame (KOPF) into the motor cortex of animals with injured CST at 35DPI using the fibre optic cannula hole, in order to trace the stimulated CST. The tracer was injected at 0.2 μl/min for a total of 1 μl, adding 5 more minutes at the end to avoid liquid spillage. 5 days were waited before sacrifice, to allow the tracer to reach the axonal terminations.

### Immunocytochemistry (ICC)

For immunocytochemistry (ICC), cells were fixed with 4% paraformaldehyde (PFA) 15 min on ice, washed and incubated in blocking solution (1% Bovine serum albumin (BSA), 0,25% Triton X-100 (0,25% Tx) in 0.1M PBS) for 1 h at RT. Primary antibody (βIII tubulin (Tuj1, 1:1000, BioLegend), c-Fos (1:200, Cell Signaling), ChR2 (1:500, Progen), mCherry (1:500, Abcam) GFP (1:500, ThermoFisher Scientific) was added to the cells in blocking solution and incubated O/N at 4 °C. After washing, the cells were incubated 1 h at RT with Alexa-Fluor-conjugated secondary antibodies (568 goat and 488 goat and donkey) and Hoechst (Sigma Aldrich).

### Tissue processing for immunohistochemistry

Anaesthetized mice were transcardially perfused with ice-cold 4% paraformaldehyde (PFA) and the spinal cord, the brain or the DRGs were dissected and allowed to post-fix in 4% PFA for 24 h at 4 °C. In peripheral experiments the sciatic nerves and the DRGs were directly dissected, as perfusion was not needed, and allowed to fix in 4% PFA for 2 h on ice. Fixed tissues were cryoprotected in 30% sucrose in PBS for 24 h at 4 °C, brains were then directly frozen and sliced at 30 μm with a freezing microtome (2000R Leica), while spinal cords and DRGs were embedded in tissue freezing medium (OCT, Sigma Aldrich), frozen and 18 μm or 10 µm slices respectively were obtained using a cryostat (CM 1900 Leica) and directly mounted. For whole mount stainings cryprotection was not needed, instead tissues were kept in 0.1M PBS.

### Immunohistochemistry (IHC)

Prior to IHC, brain slices were maintained in cryoprotection solution (30% glycerol, 30% ethylene glycol, 40% PBS). Brain IHCs were performed directly on free floating slices. Slices were blocked for 1 h at RT (10% FBS, 0.5% Tx, 0.2% gelatine, 0.2M glycine in 0.1M PBS) and incubated with primary antibody O/N at 4 °C (5% FBS, 0.5% Tx, 0.2% gelatine in 0.1M PBS; GFP (1:500, ThermoFisher Scientific). Slices were then repeatedly washed with PBS-0.5% Tx, incubated with secondary antibodies (Alexa Fluor 488 donkey; ThermoFisher Scientific) and Hoechst (Sigma Aldrich).

IHCs were performed directly on spinal cord or DRG preparations. Slides were blocked for 1 h at RT (Blocking solution: 8% BSA, 0.3% Tx in TBS) was added and incubated for 2 h at RT, followed by O/N incubation of primary antibody (GFAP (1:500, Dako); cFos (1:200, Cell Signaling); NFH (1:200; Abcam)) in 2% BSA, 0.2% Tx in TBS at 4 °C. Secondary antibodies (Alexa Fluor 488 donkey and goat and 568 goat; ThermoFisher Scientific) and Hoechst were added after washing with TTBS and incubated 1 h at RT.

Whole-mount IHCs were performed for sciatic nerves. Blocking solution (8% BSA, 1% Tx and 1/150 mαIgG (Fabs) in TBS), followed by 3 O/N of primary antibody incubation (SCG-10 (1:1000, Novus Biologicals) in 2% BSA, 0.3% Tx in TBS at 4 °C. Secondary antibodies (Alexa Fluor 488 donkey) were added after washing with TTBS and incubated 1 O/N at 4 °C.

Each preparation was subsequently mounted in Mowiol™ (Sigma Aldrich).

### Fluorescence intensity analysis

To measure c-Fos intensity, DRG neurons were immunostained for c-Fos and ChR2 and imaged at 40× magnification with an Olympus microscope IX71 using an Orca Flash 4 (Hamamatsu Photonics). Only ChR2^+^ cells were selected for this analysis. About 20 cells per well were analysed. The nuclei of the cells were selected using Hoechst counterstaining, and its mean c-Fos intensity determined by subtracting the corresponding background value to the integrated density (Corrected total cell fluorescence; CTCF), measured using ImageJ™.

### Neurite and axonal length and branching analysis

Images were taken at 10× magnification with an Olympus microscope IX71 using an Orca Flash 4 or a CX50 Olympus camera. TUJ1, mCherry or GFP was used to immunolabel neurites and axons. For DRG neurite analysis, three fields per well were analysed and the average neurite length per cell was obtained. Small diameter (< 35 µm) neurons were excluded from the analysis. In microfluidic devices, the percentage of regenerating axons normalized to the total number of axons reaching the axonal compartment was determined. For cortical neuron cultures, 8–9 fields per device were analyzed. Either average axon length or total area covered by axons was quantified. Neurite-J plugin in ImageJ™ software was used for neurite and axonal measurements (Meijering et al. 2004). Area measurements were measured using ImageJ™ software. For branching analysis, total number of neurite branches per cell was quantified using ImageJ™ software and normalized to neurite length (100 µm) for each quantified neuron.

### Sprouting quantification

To quantify the number of sprouting axons before and after the lesion, we measured the tracer fluorescence intensity on sections at 0.5 mm pre-injury and post-injury level. The spinal cord section images were divided in two different ROIs corresponding to grey matter and white matter for analysis.

Spinal cord sections of stimulated and non-stimulated Thy1-ChR2 mice at 500 µm rostral to the lesion core were obtained using a confocal microscope (LSM 800, AxioCam 503c; Zeiss) at 10× magnification. The number of double positive axons for Dextran-Alexa 594/ChR2-YFP in the grey matter at different distances from the CST in the same section was computed and normalized to the number of Dextran-traced CST.

### Nerve regeneration analysis

Regenerating sensory axons were immunolabeled with SCG10. Whole mount preparations were imaged with a confocal microscope (LSM 800, AxioCam 503c; Zeiss) at 5× magnification. 6–8 tiles and 10–15 slices were obtained per nerve and the Blue Zen™ and ImageJ™ softwares were used to reconstruct the nerve and obtain a Maximum Intensity Z-projection. The crush site was determined by phase contrast images and the number of regenerating axons at several distances from the crush was determined.

### Statistical analysis

Prism 6.0 (GraphPad™ Software) was used for statistics and graphical representation. Plotted data shows mean ± s.e.m (standard error of the mean). Normality was determined with Shapiro–Wilk test. Significant differences are indicated by arterisks (*p < 0.05; **p < 0.01; ***p < 0.005; ****p < 0.001). ANOVA followed by Bonferroni post hoc test or Student’s t-Test were used in normal distributions as opposed to Mann–Whitney or Kruskal–Wallis tests as non-parametric tests for samples that did not meet normality.

## Results

### Optogenetic stimulation of DRG neurons increases their axonal growth in vitro

We first sought to determine if optogenetic activation of adult DRG neurons could enhance axonal growth in vitro. Adult dissociated Thy1-ChR2 DRG neurons were optically stimulated and allowed to grow for 24 h.

**Fig. 1:**
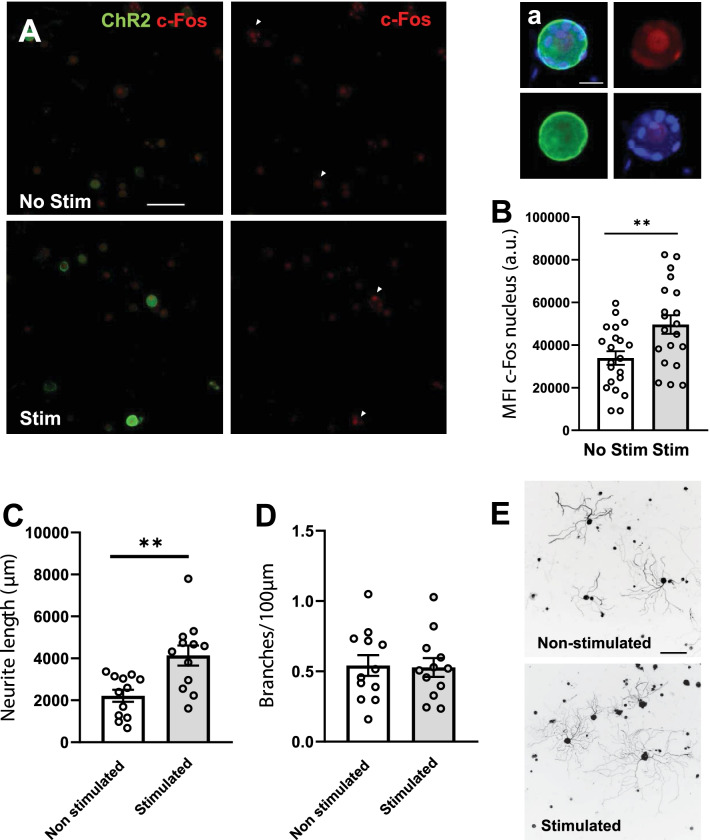
Increased neuronal activity promotes neurite outgrowth. **A** Representative images of c-Fos (red) and ChR2 (green) immunostaining. White arrows depict ChR2^+^ neurons. Scale bar: 100 μm. a. Higher magnification image of a stimulated neuron. ChR2 expression is mainly located in the membrane of large-diameter neurons. Scale bar: 20 μm. **B** c-Fos expression in the nucleus is increased just after optogenetic stimulation of Thy1-ChR2 DRG neurons (n = 20–25 cells). MFI: mean fluorescence intensity. a.u.: arbitrary units. Data are expressed as mean nuclear fluorescence intensity ± s.e.m. **p < 0.01 denotes significant differences in Student’s t-Test. **C** Stimulated neurons presented significantly higher neurite lengths when compared to non-stimulated ones. Average neurite length per neuron was determined at 24 h in vitro. Data are expressed as mean ± s.e.m. (n = 12 images; **p < 0.01 denotes significant differences in Student’s t-Test). **D** Stimulation did not significantly modify the neurite branching. Average number of branches for each 100 µm was determined at 24 h in vitro. Data are expressed as mean ± s.e.m (n = 12 images). **E** Representative images of Tuj-1 staining used to analyze neurite length. Scale bar: 200 μm

Optical stimulation effectively increased neuronal activity in DRG neurons, as displayed by the increased levels of c-Fos staining after stimulation (49,627 ± 4373 a.u. of nuclear CTCF intensity for stimulated DRGs versus 33,897 ± 3212 a.u. of nuclear CTCF intensity for non-stimulated; *p* = 0.0058 Student’s t-Test) (Fig. 1A, B).

Optically stimulated neurons showed increased neurite outgrowth (4053 ± 433.1 µm) after 24 h of culture when compared to non- stimulated ones (2029 ± 647.7 µm; *p* = 0.0267 Student’s t-Test), but did not show any differences in neurite branching (non-stimulated: 0.5418 ± 0.0735 branches/100 µm; stimulated: 0.5277 ± 0.0668 branches/100 µm; *p* = 0.8884 Student’s t-Test) (Fig. 1C–E).

### Chemogenetic stimulation of DRG neurons improves their regenerative capacity after in vitro axotomy and sciatic nerve crush

**Fig. 2:**
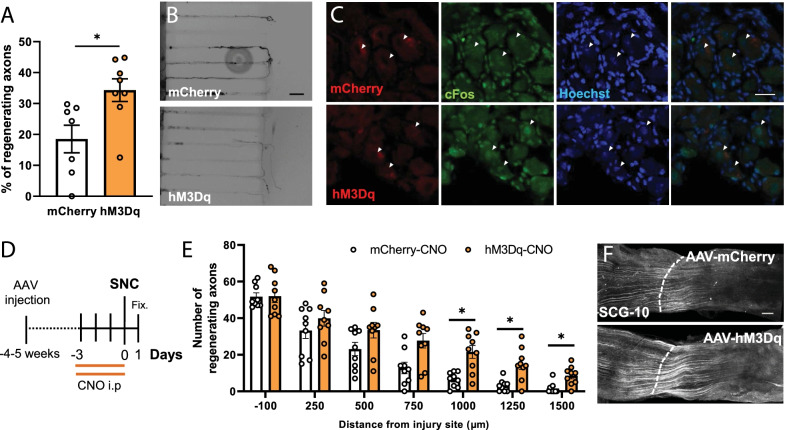
Chemogenetic stimulation induced PNS regeneration. **A** Chemogenetically stimulating DRG neurons in vitro 6 h after axotomy resulted in regeneration promotion. Data are expressed as the mean percentage of infected regenerating axons compared to the total of infected axons reaching the axonal compartment ± s.e.m (n = 7–8 axonal compartments). *p < 0.05 denotes significant differences in Student’s t-Test. **B** Representative images of hM3Dq/mCherry^+^ axons. Scale bar: 50 μm. **C** c-Fos nuclear staining is observed in hM3Dq infected DRG neurons (white arrows) 1 h after CNO injection, but not in mCherry^+^ DRG neurons after the same treatment. Scale bar: 25 µm. **D** Schematic timeline of the experiment. **E** The number of regenerating sensory axons (SCG-10^+^) in stimulated nerves (hM3Dq-CNO) is increased in all assessed distances compared to the non-stimulated (mCherry-CNO) reaching statistical significance in long distances. Data are expressed as mean ± s.e.m. at each distance from the injury site (n = 9 sciatic nerves). *p < 0.05 denotes significant differences in ANOVA followed by Bonferroni test. **F** SCG-10 immunostaining of mCherry or hM3Dq infected sciatic nerves 24 h after SNC. Dotted lines indicate the injury site. Scale bar: 200 μm

We then wanted to test if this increased growth capacity also translated in regenerative capacities after axotomy. To this aim we first evaluated regenerative capacity of DRG neurons in vitro using microfluidic assisted axotomy and chemogenetic stimulation. DRG neurons were seeded in custom microfluidic devices as previously described, and hM3Dq or mCherry expression was induced by infection with AAVs. Axons were allowed to grow for few days through the microchannels until they reached the axonal chamber and then we performed a vacuum assisted axotomy. CNO was administered 6 h after axotomy. Chemogenetic stimulation resulted in enhanced axonal regeneration (*p* = 0.0165; Student’s t-Test) when compared to non-stimulated mCherry controls (Fig. 2A, B).

We then proceeded to validate if the in vitro results in fact translated to enhanced axon regeneration in vivo after PNS injury. Due to the difficulty to apply light in the DRGs for long periods of time in awake animals, we used chemogenetics for activity control. We first verified if DRGs were transduced in vivo. AAV5-hSyn-mCherry or AAV5-hSyn-hM3Dq-mCherry were carefully injected into the sciatic nerve and mCherry expression was examined 4–5 weeks later. Both vectors transduced DRG neurons with similar efficiency (~ 35% of total DRG neurons; ~ 65% of large diameter (> 35 µm) neurons (Additional file 1: Figure S1A, B). mCherry expression was mainly localized in the soma of large diameter NFH^+^ DRG neurons (Additional file 1: Figure S1C).

To verify the activation of DRG neurons in vivo after chemogenetic stimulation, we performed cFos staining 1 h after CNO administration, and observed increased nuclear cFos signal only in hM3Dq animals compared to mCherry controls (Fig. 2C).

To assess the effects of neuronal activity on PNS regeneration, animals were administered with 5 mg/kg CNO twice a day for 4 days starting 3 days before performing a bilateral SNC to guarantee four consecutive stimulation days (Fig. 2D). 24 h after injury hM3Dq stimulated animals showed increased number of regenerating sensory axons (SCG10^+^) at further distances (> 750 µm) when compared to mCherry controls. The two-way ANOVA showed a significant effect of the distance (*p* < 0.0001) and stimulation (*p* = 0.0145) as well as their interaction (*p* = 0.0101). At 750 µm from the injury stimulated animals showed 27.67 ± 4.19 axons versus 12.78 ± 3.34 axons on non-stimulated animals (*p* = 0.0694 Bonferroni post Hoc test), at 1000 µm from the injury stimulated animals showed 21.56 ± 3.72 axons versus 6.44 ± 1.43 axons on non-stimulated animals (*p* = 0.0163 Bonferroni post hoc test), at 1250 µm from the injury stimulated animals showed 14.89 ± 3.12 axons versus 3.11 ± 1.14 axons on non-stimulated animals (*p* = 0.0261 Bonferroni post hoc test), and at 1500 µm from the injury stimulated animals showed 8.56 ± 1.81 axons versus 1.56 ± 1.06 axons on non-stimulated animals (*p* = 0.0259 Bonferroni post hoc test) (Fig. 2E, F).

### Optogenetic stimulation of DRG neurons increases their axonal growth in vitro, only on permissive substrates

To assess the translatability of these results into a CNS injury paradigm, we first checked whether well-defined CNS inhibitory signals, such as CSPGs, affected the activity-induced increased axonal growth. To this aim, we cultured adult DRG neurons on both permissive (Laminin) substrates and on different concentrations of inhibitory (CSPG) substrates and subjected them to optogenetic stimulation.

**Fig. 3:**
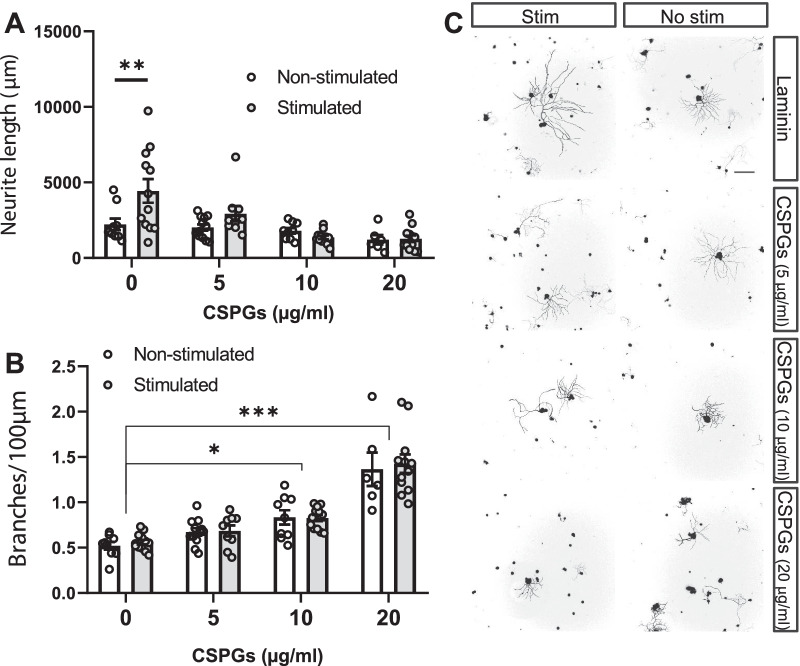
Neuronal activity induces growth on permissive but not inhibitory substrates. **A** Neurite outgrowth was significantly increased in optogeneticallty stimulated DRG neurons over growth-permissive substrates (0; laminin), but not over different concentrations (5, 10, 20 μg/ml) of non-permissive substrates (CSPGs). Average neurite length per neuron was determined at 24 h in vitro. Data are expressed as mean neurite length per cell ± s.e.m (n = 7–12 images). **p < 0.01 denotes significant differences in ANOVA followed by Bonferroni test. **B** Neurite branching is increased dose-dependently by CSPGs but is not affected by neuronal stimulation. Average number of branches for each 100 µm was determined at 24 h in vitro. Data are expressed as mean ± s.e.m (n = 7–12 images; *p < 0.05; ***p < 0.005 denote significant differences vs respective 0 µg/ml condition in ANOVA followed by Bonferroni test). **C** Representative images of Tuj-1 staining used to analyze neurite length. Scale bar: 200 μm

As previously observed (Fig. 1C–E), optogenetic stimulation increased the neurite outgrowth in permissive substrates (3761 ± 290.8 µm for stimulated DRGs versus 2735 ± 162.9 µm for non-stimulated DRGs), but not on neurons seeded on any concentration of CSPGs, including the 5 µg/ml concentration, that did not reduce basal growth (Fig. 3A, C), the two-way ANOVA did not show a significant effect from the stimulation (*p* = 0.4785), while showing a significant effect of the substrate (*p* < 0.0001) and their interaction (*p* = 0.0398), highlighting the blockage of the stimulation effects in the presence of CSPGs.

In terms of neurite branching (Fig. 3B, C), the two-way ANOVA revealed an effect of the CSPG (*p* < 0.0001) but neither from the stimulation (*p* = 0.6088) nor their interaction (*p* = 0.9682). Bonferroni post-hoc comparisons further exposed differences between the laminin groups and the 10 and 20 µg/ml CSPG groups, both in the stimulated and non-stimulated conditions, while all intercomparisons between stimulation conditions did not show any differences.

### Optogenetic stimulation of CNS neurons increases their axonal regeneration in vitro

**Fig. 4:**
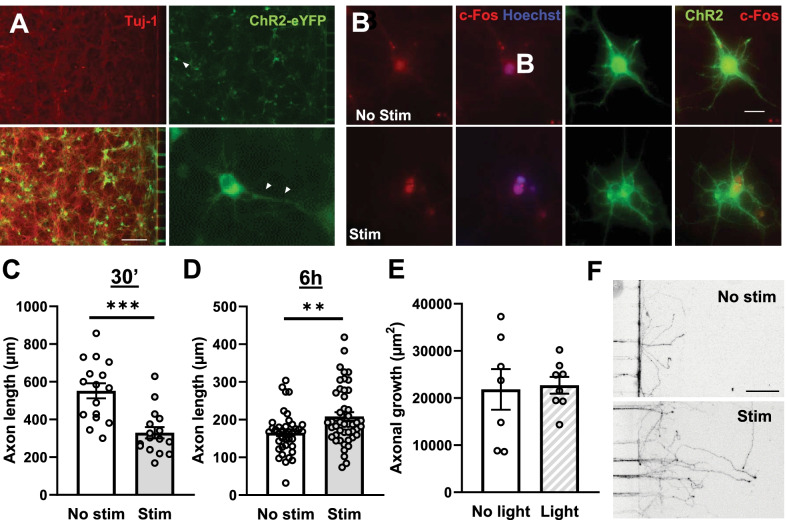
Optogenetic stimulation of cortical neurons after axotomy. **A** Cortical neurons (Tuj-1) express ChR2-eYFP after LV-ChR2 infection. White arrows in the high magnification image indicate neuritic expression of ChR2. Scale bar: 250 μm. **B** Optogenetic stimulation increased the expression of c-Fos in cortical neurons. Representative images of c-Fos (red) and ChR2 (green) immunostaining. Scale bar: 20 μm. **C**, **D** Optogenetic stimulation of cortical neurons 30 min after axotomy (**C**) resulted in reduced axon regeneration, while delivering the stimulation 6 h after axotomy (**D**) increased axon regeneration. Individual ChR2^+^ axon lengths were quantified (**C**: n = 16 images; **D**: n = 37–43 images). Data are expressed as mean ± s.e.m. ***p < 0.001 denotes significant differences in Student’s t-Test; **p < 0.01 denotes significant differences in Mann Whitney test. **E** Optical stimulation (470 nm light) of GFP-expressing cortical neurons did not induce any changes in axonal regeneration. Total growth area/microchannel was computed (n = 7–8 images). Data are expressed as mean ± s.e.m. **F** Representative images of GFP/YFP staining used to analyze axon regeneration when stimulation is applied 6 h after axotomy. Scale bar: 200 μm

We then wanted to test if this intrinsic increased regeneration capacity was also present in CNS neurons. To this aim we evaluated the regenerative capacity of cortical neurons in vitro after optogenetic stimulation. Embryonic cortical neurons were seeded in custom microfluidic devices as previously described, ChR2 expression was induced by infection with LV-ChR2 (Fig. 4A), and activation of neurons upon stimulation was assessed by cFos staining immediately after stimulation (Fig. 4B). Axons were allowed to grow for few days through the microchannels until they reached the other chamber, when a vacuum assisted axotomy was performed. Optogenetic stimulation of cortical neurons decreased axonal regrowth when the stimulation was delivered 30 min after the axotomy (Fig. 4C). In contrast, when applied 6 h after the axotomy, optogenetic stimulation resulted in an increase in axonal regrowth when compared to non-stimulated ones (*p* = 0.0056; Mann–Whitney test; Fig. 4D–F). To control the intrinsic effect of blue light on neuronal growth, we applied the light stimulation pattern on non-stimulable (GFP controls) embryonic cortical neurons and observed no difference in axonal growth between illuminated and non-illuminated samples (Fig. 4E).

### Optogenetic stimulation of cortical motor neurons does not improve sensorimotor performance after SCI

**Fig. 5:**
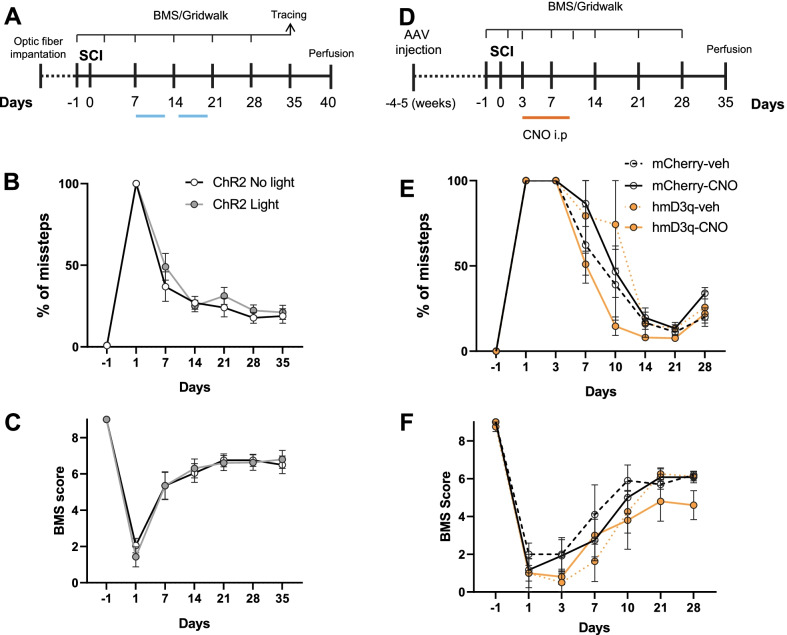
Increasing neuronal activity does not induce recovery after CNS injury. **A** Timeline of the CST injury and stimulation experiments. **B**, **C** % of missteps in the gridwalk test (**B**) (n = 8–9 mice per group for each timepoint) and BMS score (**C**) (n = 10 mice per group for each timepoint) show no differences in sensorimotor recovery in stimulated Thy1-ChR2 (ChR2 Light) mice when compared to non-stimulated (ChR2 No light) after CST injury. **D** Timeline of the DCA and stimulation experiments. **E**, **F** Chemogenetically stimulated animals (hM3Dq-CNO) show similar sensorimotor function recovery to non-stimulated ones (mCherry-CNO, mCherry-veh, hM3Dq-veh) after DCA as seen by the gridwalk (**E**) (n = 3–5 mice per group for each timepoint) and BMS (**F**) (n = 5–6 mice per group for each timepoint) tests. Represented data correspond to the BMS score and % of missteps in the gridwalk. Data are expressed as mean ± s.e.m. ANOVA followed by Bonferroni post-hoctest

To test if these results further translated to a better motor performance after SCI in vivo, we implanted optic fibre cannulas on Thy1-ChR2 animals. These animals, have the cortical expression of ChR2 predominantly concentrated in the layer V, where corticospinal motor projecting neurons lay (Additional file 2: Figure S2A). After recovery, animals were subjected to a CST axotomy, and optical stimulation was performed daily from day 7 after injury (Fig. 5A). Stimulated animals did not show any improvement in their motor performance on the Gridwalk (Fig. 5B) or the BMS (Fig. 5C) when compared to non-stimulated controls. For each test evaluated, the two-way ANOVA showed a significant effect of the time (BMS *p* < 0.0001; Gridwalk *p* < 0.0001) but neither from the stimulation (BMS *p* = 0.7201; Gridwalk *p* = 0.1308) nor from their interaction (BMS *p* = 0.9905; Gridwalk *p* = 0.0707). In all tests, non-significant changes were observed for each timepoint when compared stimulated versus non-stimulated mice (Bonferroni multiple comparisons test).

These results indicate that while in vitro CNS neurons have the ability to increase their axonal growth capacity when stimulated, after CNS injury in vivo there are inhibitory signals, such as CSPGs, that block this enhanced regeneration*.*

### Chemogenetic stimulation of DRG neurons does not improve sensorimotor performance after SCI

To further test whether the lack of in vivo functional recovery was due to an intrinsic inability to regenerate from corticospinal neurons or due to the presence of an extrinsic inhibitory signal in the CNS injury, we tested the effects of chemogenetic stimulation on DRG neurons after a dorsal hemisection of the spinal cord in vivo*.* Similarly to what was done for the PNS injury, AAV5-hSyn-mCherry or AAV5-hSyn-hM3Dq-mCherry were carefully injected into the sciatic nerve to induce its expression on the DRG (Additional file 1: Figure S1A–C). 4–5 weeks later animals were subjected to a dorsal hemisection, and 3 days after injury animals received i.p. injections of CNO (5 mg/kg b.w.) twice a day for 7 days (Fig. 5D). As observed for the cortical stimulation, stimulated animals did not show any improvement in their sensorimotor performance on the Gridwalk (Fig. 5E) or the BMS (Fig. 5F) when compared to mCherry-veh, mCherry-CNO or hM3Dq-veh controls. For each test evaluated, the two-way ANOVA showed a significant effect of the time (BMS *p* < 0.0001; Gridwalk *p* < 0.0001) but neither from the stimulation (BMS *p* = 0.2289; Gridwalk *p* = 0.0694) nor from their interaction (BMS *p* = 0.3614; Gridwalk *p* = 0.0785). In all tests, no significant changes were observed for each timepoint when compared stimulated versus non-stimulated mice (Bonferroni multiple comparisons).

These results again suggest the presence of an inhibitory environment after CNS injury that blocks the regeneration induced by neuronal stimulation. In contrast after PNS injury where no inhibitory environment is present, stimulation did induce enhanced regeneration.

### Optogenetic stimulation of corticospinal motor neurons increases prelesion sprouting but does not induce regeneration across the lesion

**Fig. 6:**
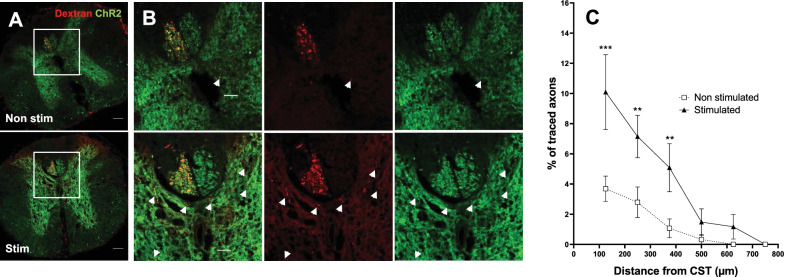
Neuronal stimulation induces axonal sprouting before the lesion. **A** Representative images of traced (Dextran-Alexa594) CST and ChR2-YFP in stimulated and non-stimulated Thy1-ChR2 mice in spinal cord sections 500 μm rostral to the lesion core. Scale bar: 100 μm. **B** Close-up higher magnification images from indicated regions on **A**. White arrows indicate double-positive Dextran-Alexa 594/ChR2-YFP sprouted axons. Scale bar: 50 μm. **C** The number of double-positive Dextran-Alexa 594/ChR2-YFP sprouted axons in the grey matter as a normalized ratio of Dextran-Alexa 594-traced CST axons shows a significantly higher percentage of sprouted axons in stimulated animals at distances up to 400 µm laterally and ventrally from CST. Data are expressed as mean ± s.e.m. at each distance from the CST (n = 4 mice) ***p < 0.001; **p < 0.01 denote significant differences in ANOVA followed by Bonferroni test

Since activity dependant therapies have been shown to increase lateral sprouting and plasticity (Carmel et al. 2010, 2013, 2014; Sánchez-Ventura et al. 2021; Goldshmit et al. 2008; Engesser-Cesar et al. 2007), we sought to investigate if our stimulation paradigm increased the sprouting of injured axons in areas before the lesion. To this aim, we analysed the spinal cords of stimulated Thy1-ChR2 mice after tracing their corticospinal tracts. Interestingly, we found that optogenetic stimulated animals did show more axonal tracing in the grey matter (33.60 ± 1.13 a.u. of CTCF intensity for stimulated animals versus 23.27 ± 0.89 a.u. of CTCF intensity on non-stimulated animals; *p* = 0.0015 Student’s t-Test) in segments before (− 0.5 mm) the injury, but not beyond (+ 0.5 mm) the injury (14.61 ± 1.21 a.u. of CTCF intensity for stimulated animals versus 12.80 ± 0.47 a.u. of CTCF intensity on non-stimulated animals; *p* = 0.30328 Student’s t-Test) (Additional file 2: Figure S2B, C). Additionally, we also quantified the number of sprouted axons in the grey matter as a ratio from traced CST axons (in order to normalize the tracing efficiency between animals). Interestingly we found that stimulated animals showed a significantly higher percentage of traced sprouts up until 400 µm ventrally and laterally from the CST (Fig. 6A–C). The two-way ANOVA showed a significant effect of the distance (*p* < 0.0001), the stimulation (*p* = 0.0401) as well as their interaction (*p* = 0.0013). These findings suggest that the positive effects of modulation of the activity on the axonal regrowth are inhibited by CNS inhibitory signals present in the injury.

## Discussion

Neuronal activity-triggered plasticity is commonly recognized as the main component of recovery in current activity-based therapies (Carulli et al. 2011; Hogan et al. 2020), however, this is mainly built on therapies that use exercise or electrical stimulation to induce neuronal activity (as in Carmel et al. 2010, 2013, 2014; Sánchez-Ventura et al. 2021; Goldshmit et al. 2008; Engesser-Cesar et al. 2007). Even so, even though these approaches increase neuronal activity, they do so without cellular specificity, hindering the identification of underlying cellular and molecular mechanisms induced by neuronal activity itself.

Taking advantage of the cellular specificity of optogenetics and chemogenetics (Deisseroth 2015; Sternson and Roth 2014) we performed different experiments to assess the specific cellular effects of neuronal activity on axonal growth using different in vitro and in vivo neuronal models.

Consistent with previous works (Sala-Jarque et al. 2020; Jaiswal and English 2017; Ward et al. 2016; Park et al. 2015), we showed that increasing neuronal activity on DRG sensory neurons enhanced axonal growth in non-inhibitory substrates in vitro and in vivo after peripheral injury. Additionally, and still accordingly to previous studies (Ward et al. 2018), we also demonstrated these effects in embryonic cortical cells in vitro*,* highlighting the presence of similar mechanisms among different neuronal types.

It is dependent on the timing after injury and the pulse frequency important to mention that before the achievement of these results, a fine tuning of the stimulation protocol was needed. Axonal growth capacities showed to be highly during the stimulation (data not shown), highlighting the fact that neuronal activity needs, not only to be stimulated, but to be finely regulated in order to promote the desired outcomes. For instance, high frequency stimulations (> 20 Hz in 1 s trains, or > 10 Hz in continuous stimulation) reduced the axonal growth in vitro*,* and induced seizures in vivo. This has also been emphasized in previous studies indicating activity-dependant effects on gene expression (Tyssowski et al. 2018; Lee et al. 2017; Miyasaka and Yamamoto 2021) and plasticity (reviewed in Fauth and Tetzlaff (2016)).

Meanwhile, timing of stimulation after injury seems to be very relevant as well, as stimulating 30 min after axotomy results in a reduction of axonal growth compared to non-stimulated controls. This might be caused by the ionic imbalance generated by membrane disruption after axonal injury, that leads to exaggerated Ca^2+^ influx. Membrane sealing and restoring the ionic balance and permeability occurs early after injury, but maintaining neuronal depolarization during this period leads to the activation of some voltage gated cation channels that have been shown to inhibit growth (Goganau et al. 2018; Enes et al. 2010; Tedeschi et al. 2016). In accordance, little changes in the stimulation patterns might lead to opposed effects, therefore we used optogenetics whenever possible, as our method of choice to stimulate activity, since it allowed us a higher temporal resolution (Rost et al. 2017). Nonetheless, the anatomical setting of the DRGs impeded the implantation of a permanent optic fiber cannula, and therefore did not allow us to perform awake stimulations. Thus, as this would have compromised the wellbeing of the animals, therefore, we opted for a chemogenetic approach for in vivo DRG stimulation. Effectively, this method rendered similar results on neurons as those observed in vitro with optogenetics, resulting in enhanced regeneration. For this experiment we delivered the chemogenetic agonist (CNO) before the injury (as a preconditioning to allow enough stimulation days in this setting), leading to similar effects as those observed in previous studies using enriched environment (Hutson et al. 2019) or electrical stimulation (Goganau et al. 2018; Udina et al. 2008; Senger et al. 2018) as a preconditioning.

Considering that our in vitro and PNS injury results have shown comparable regeneration outcomes than those of electrical stimulations (Ahlborn et al. 2007; Al-Majed et al. 2004), we believe that neuronal activity itself might be responsible, at least partially, of these effects on axonal growth.

However, when we tested this paradigm in an in vivo model of SCI, we found that optogenetic stimulation of spinal-projecting cortical neurons did not promote the expected functional recovery after SCI, contrarily to what other studies observed using electrical stimulation (Carmel et al. 2010, 2013, 2014), or exercise (Goldshmit et al. 2008; Engesser-Cesar et al. 2007; Al-Majed et al. 2000).

We then focused on clarifying whether our model did not induce any kind of axonal regeneration, or it did, but was insufficient to promote recovery. In that sense, an in vivo CNS model of injury implies the presence of a number of factors absent in our previous experiments, including both the presence of an intrinsic lack of regenerative capacity as well as the presence of extrinsic inhibitory substrates for regeneration (Loy et al. 2018; Bradbury and Burnside 2019; Mesquida-Veny et al. 2021). Accordingly, we performed complementing in vitro experiments, in which we found that stimulated neurons displayed increased axonal growth in permissive substrates (laminin), but not in CNS growth inhibitory substrates (CSPGs). Additionally, when we performed further histological characterization, we found stimulated neurons showed increased axonal growth and sprouting in the rostral vicinity of the injury, especially in the grey matter of the SC, but not caudally, across or beyond the injury. While we cannot exclude that the lack of regeneration across the injury might be due, at least in part, to the intrinsic low regenerative capacity of corticospinal neurons, our data suggests that neuronal activity stimulates the axonal growth capacity of these neurons, as seen in embryonic cortical neurons in vitro, through a mechanism that cannot overcome the repressive signalling of growth-inhibitory molecules, such as CSPGs. Additionally, in vivo injured stimulated neurons start growing through uninjured areas of the SC, similarly to what is described in other studies with classical stimulation approaches (Carmel et al. 2010, 2013, 2014), where injured and uninjured axons grow axonal processes searching for spared intraspinal circuitry, forming new connections that will eventually restore the lost connections through neuronal plasticity, bypassing the injury (Courtine et al. 2019). Interestingly, classical non-specific stimulations such as epidural electrical stimulation or rehabilitation led to stimulation of intraspinal circuits, involving spinal interneurons and motoneurons, this stimulation in turn promotes the reorganization and activation of this circuitry (Wagner et al. 2018). Whether the lack of these functional effects with our paradigm was due to a short stimulation period, or that it lacked the direct stimulation of this intraspinal circuitry and thus did not induce the plasticity needed, remains still unanswered. In this direction, a recent study also observed a blockage of activity-induced axonal growth in the presence of inhibitory-substrates after cellular specific stimulation (Wu et al. 2020). In this study, functional recovery after CNS injury was achieved after combining chemogenetic stimulation and Chondroitinase ABC (ChABC, a CSPG degrading enzyme) administration (Wu et al. 2020), but surprisingly only after 6 weeks of continuous combined treatment. In contrast to both this work and our results, both chemogenetic stimulation and visual stimulation induce functional regeneration after optic nerve crush, a CNS injury model (Lim et al. 2016). Importantly however, the expression of the different chemogenetic receptors in this study was induced by intravitreal injection of the vectors, meaning that retinal interneurons, including amacrine and bipolar cells, will most likely be expressing the channels and be subjected to stimulation upon agonist administration. This paradigm resembles to that of electrical stimulation, considering activation of this retinal interneurons might be inducing neuronal plasticity and facilitating functional recovery.

Seminal works from the early 90 s decade first showed the inhibitory effect of CSPGs in embryonic DRG and retinal ganglion cells (García-Alías et al. 2009; Wang et al. 2011). Later works have corroborated the presence of CSPGs in the injury core and glial scar as one of the main inhibitors of regeneration in the CNS after injury, which opened the possibility to use their degradation as therapeutical strategies for CNS injuries (Griffin et al. 2020; Griffin and Bradke 2020). Additionally, there is plenty of evidence that neuronal activity itself is key in promoting regeneration and recovery, however, the presence of growth inhibitory substrates in the injured CNS that limit regeneration, might as well be limiting the success of activity-based therapies. In line with this, combining functional rehabilitation or exercise with CSPG degrading therapies (including ChABC (Misonou et al. 2004; Romer et al. 2016) or ADAMTS4 (a disintegrin and metalloproteinase with thrombospondin motifs 4) (Shim and Ming 2010) leads to synergistic effects, even when applied at chronic time-points (Romer et al. 2016). These reinforce the concept that multifactorial therapies are the way to go in order to tackle the different aspects of the pathophysiology of SCIs (Bas-Orth et al. 2017).

Although neither our experiments nor other studies (Wu et al. 2020), demonstrate the presence of an activity-triggered transcriptional switch, more chronic stimulations could be leading to it, although with our current knowledge, more local and transient cellular mechanisms seem to be responsible of the growth differences observed with stimulation of activity specifically at a cellular level. For example, neuronal activity changes the excitability of neurons through reorganization of several ionic channels (Enes et al. 2010; Segarra-Mondejar et al. 2018; Hu et al. 2008), this can in turn influence growth, as a result of intracellular ionic adjustments and their downstream associated signalling (Tedeschi et al. 2016; Eisdorfer et al. 2020), or even translational changes (Enes et al. 2010). Neuronal activity can also alter neuronal metabolism, increasing glycolysis, and lipid synthesis and integration to the membrane, a cellular process essential during axonal extension (Alilain et al. 2008; Asboth et al. 2018). Another important cellular component altered by activity is the cytoskeleton and its dynamics, for instance, neuronal activity has been shown to increase dendritic spine microtubule polymerization (Deng et al. 2021). Accordingly, chemogenetic stimulation increases microtubule dynamics in the distal axonal portion, by reducing tubulin acetylation and increasing tyrosination in this region (Wu et al. 2020). These mechanisms may not be exclusive, on the contrary, they are likely to take place all together facilitating activity-induced axonal growth.

On the other hand, success of activity-based therapies does not depend solely on the cellular effects of activity modulations, instead, these therapies, that target activity in a more general and chronic way (as different neurons or even circuits are stimulated simultaneously), benefit from a raised general excitability that seems to be the ultimate responsible for the plasticity and reorganization that leads to recovery (Courtine et al. 2019). In that direction, rehabilitation and electrical stimulation after injury promote the formation of new synapses in the spinal cord (Mesquida-Veny et al. 2021; McPherson et al. 2015), while respiratory function recovery after optogenetic stimulation is also attributed to synaptic plasticity (Mishra et al. 2017). Moreover, a recent study applying rehabilitation together with electrochemical stimulation showed improved recovery, which is credited to cortico-reticulo-spinal circuit remodelling (Asboth et al. 2018). Additionally, sprouting of spared axons, instead of injured ones, is also responsible for recovery after exercise and/or electrical stimulation (Carmel et al. 2010, 2013, 2014; Goldshmit et al. 2008; Engesser-Cesar et al. 2007). In agreement to that, success of these approaches is only significant in incomplete injuries, and greater as more tissue is spared. Incomplete injuries might also help explaining why in our model we did not observe recovery after optogenetic stimulation of the motor cortex while others did (Deng et al. 2021), as different spinal injuries were used. Compellingly, compression injury leaves more uninjured tissue and spinal tracts than our injury, that axotomizes all the dorsal tracts, including the main component of the CST in mice. Besides, complete injuries also present larger glial scars, and therefore greater accumulation of growth inhibitory molecules, which translates in larger distances and hurdles to overcome or bypass to achieve functional connections, together with a greater loss of intraspinal circuits.

These observations strengthen the view that current activity-based therapies stimulate plasticity on top of inducing regeneration in specifically stimulated neurons, but probably without inducing long-lasting cellular reprogramming. This plasticity results from activity-triggered local changes, therefore prolonged stimulations are more effective increasing growth or regeneration (Courtine et al. 2019). This is also evidenced by the presence of functional recovery after 12, but not after 4 weeks of chemogenetic stimulation (Wu et al. 2020). Therapeutically, holistic more unspecific approaches, have a more potent effect than cellular specific stimulations alone, as evidenced by enriched environment compared to chemogenetic stimulation, which presents a lower rate of regeneration (Hutson et al. 2019). Studies have also shown that this activity-induced plasticity can be accelerated and improved by linking the activation of different relays topographically in a system (Wagner et al. 2018; McPherson et al. 2015; Mishra et al. 2017). These systems are however more challenging to define underlying mechanisms and understand the true nature of the gains of these therapies.

## Conclusions

We found that specific cellular stimulation of neuronal activity induced axonal growth, but only in the absence of inhibitory substrates in vitro*,* or in vivo in the PNS. Likewise, our approach seems to be therapeutically less efficient in enhancing recovery than other more chronic or general stimulations. This also suggests and strengthens the idea that activity-based therapies succeed because of local transient cellular changes coupled with neuronal plasticity, rather than resulting in a cellular reprogramming of growth capacities, and because of that, longer stimulation periods elicit more robust responses.

## Supplementary Information

**Additional file 1: Figure S1.** AAV-mCherry and AAV-hM3Dq infection of DRG neurrons. A. Images of mCherry expression in AAV-mCherry and AAV-hM3Dq infected DRG neurons 4–5 weeks after viral injection. Scale bar: 100 μm. B. Large-diameter DRG neurons are preferentially infected. % of large-diameter (diameter > 35 μm) infected neurons was analyzed (n = 7–9 DRGs). Data are expressed as mean ± s.e.m. C. Both AAVs infect NFH^+^ cells. Scale bar: 30 μm.

**Additional file 2: Figure S2.** ChR2 expression and growth after CST axotomy in Thy1-ChR2 mice. A. While some layer II–III neurons express ChR2, expression is concentrated in layer V neurons. Scale bar: 100 μm. B. Schematic representation of the quantification in C. The spinal cord was divided in grey matter and white matter for analysis. C. Tracer intensity quantification of injured stimulated and non-stimulated Thy1-ChR2 mice at 0.5 mm pre-injury and post-injury. Optogenetic stimulation of the CST resulted in increased sprouting in the grey matter pre-injury, but not post-injury. a.u.: arbitrary units. Data are expressed as mean tracer intensity ± s.e.m (n = 5 spinal cords). **p < 0.01 denotes significant differences in Student’s t-Test.

## Data Availability

All data generated or analysed during this study are included in this published article or available from the corresponding author on reasonable request.

## Abbreviations

AAV: Adeno associated virus
ADAMTS4: A disintegrin and metalloproteinase with thrombospondin motifs 4
ANOVA: Analysis of Variance
BMS: Basso Mouse Scale
BSA: Bovine serum albumin
ChABC: Chondroitinase ABC
ChR2: Channelrhodopsin 2
CNS: Central nervous system
CSMN: Corticospinal motor neurons
CSPG: Chondroitin sulfate proteoglycans
CST: Corticospinal tract
CTCF: Corrected total cell fluorescence
DCA: Dorsal column axotomy
DIV: Days in vitro
DMEM: Dulbecco’s Modified Eagle Medium
DPI: Days post injury
DRG: Dorsal root ganglia
FBS: Fetal bovine serum
GFAP: Glial fibrillary acidic protein
GFP: Green fluorescent protein
ICC: Immunocytochemistry
IHC: Immunohistochemistry
LED: Light emitting diode
LV: Lentivirus
MW: Molecular weight
NA: Numerical aperture
NFH: Neurofilament heavy
O/N: Overnight
PBS: Phosphate buffered saline
PFA: Paraformaldehyde
ROI: Region of interest
Rpm: Revolutions per minute
RT: Room temperature
TBS: Tris buffered saline
TTBS: Tween-Tris buffered saline
WT: Wild type
